# A group II metabotropic glutamate receptor 3 (mGlu3, GRM3) isoform implicated in schizophrenia interacts with canonical mGlu3 and reduces ligand binding

**DOI:** 10.1177/0269881117715597

**Published:** 2017-06-28

**Authors:** Aintzane García-Bea, Isabel Bermudez, Paul J Harrison, Tracy A Lane

**Affiliations:** 1 Department of Psychiatry, University of Oxford, Warneford Hospital, UK; 2 Oxford Brookes University, Oxford, UK; 3 Oxford Health NHS Foundation Trust, Warneford Hospital, Oxford, UK

**Keywords:** Metabotropic glutamate receptor, alternative splicing, G protein-coupled receptor, isoform, schizophrenia

## Abstract

As well as being expressed as a full-length transcript, the group II metabotropic glutamate receptor 3 (GRM3, mGlu3) gene is expressed as an mRNA isoform which lacks exon 4 (GRM3Δ4) and which is predicted to encode a protein with a novel C terminus (called mGlu3Δ4). This variant may contribute to the mechanism by which GRM3 acts as a schizophrenia risk gene. However, little is known about the properties or function of mGlu3Δ4. Here, using transiently transfected HEK293T/17 cells, we confirm that GRM3Δ4 cDNA is translated, with mGlu3Δ4 existing as a homodimer as well as a monomer, and localizing primarily to cell membranes including the plasma membrane. Co-immunoprecipitation shows that mGlu3Δ4 interacts with canonical mGlu3. mGlu3Δ4 does not bind the mGlu2/3 antagonist [^3^H]LY341495, but the presence of mGlu3Δ4 reduces binding of [^3^H]LY341495 to mGlu3, paralleled by a decrease in the abundance of membrane-associated mGlu3. These experiments indicate that mGlu3Δ4 may negatively modulate mGlu3, and thereby impact on the roles of GRM3/mGlu3 in schizophrenia and as a therapeutic target.

## Introduction

The group II metabotropic glutamate receptor 3 (mGlu3) is a seven trans-membrane domain, G protein-coupled receptor (GPCR) encoded by the GRM3 gene on chromosome 7q21.1-2. It is negatively coupled to adenylate cyclase, and acts in part as an inhibitory autoreceptor, influencing synaptic plasticity and many other aspects of brain function (Niswender and Conn, 2010). A role for the receptor has been advocated in the pathophysiology and therapy of many neuropsychiatric disorders, including mood and anxiety disorders (e.g. Kandaswamy et al., 2013; Kim et al., 2014; Swanson et al., 2005). However, it is schizophrenia in which mGlu3 is most strongly implicated (Harrison et al., 2008; Moreno et al., 2009). Involvement was initially suggested by the finding that mGlu2/3 agonism could reverse behavioural and cognitive deficits caused by NMDA receptor antagonism, a widely used model of the disorder, both in rodents and humans (Krystal et al., 2005; Moghaddam and Adams, 1998). These findings were complemented by candidate gene studies showing associations of GRM3 single nucleotide polymorphisms (SNPs) with schizophrenia and relevant endophenotypes (Egan et al., 2004; Tan et al., 2007). The evidence became more compelling when the GRM3 locus was found to be genome-wide significant for schizophrenia (Schizophrenia Working Group of the Psychiatric Genomics Consortium, 2014). Thus, although promising clinical trial evidence that mGlu2/3 agonism is an effective antipsychotic strategy (Patil et al., 2007) has not been confirmed (Kinon et al., 2015), there remains considerable interest in the mechanisms by which GRM3/mGlu3 may contribute to schizophrenia and its treatment.

**Figure 1.**
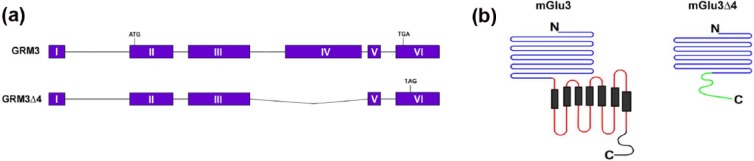
Schematic of mGlu3Δ4 and its relationship to canonical mGlu3. (a) Schematic of the GRM3 locus. The full-length receptor derives from a six exon transcript (GRM3), with the seven transmembrane domain region encoded by exon IV. The start codon is in exon II and the stop codon in exon VI. GRM3Δ4 is a transcript which lacks exon 4, resulting in a frameshift leading to novel C terminus. (b) Full-length mGlu3, showing the large extracellular N terminal region (blue), the transmembrane domain (red, with black rectangles) and the intracellular C terminus (black). The ligand binding domain lies within the extracellular region, from amino acids 25-508. mGlu3Δ4 has the same N-terminal 441 amino acids, but lacks the rest of the extracellular domain and the transmembrane domain, and with a shorter, novel C terminus (green). The amino acid sequence of the novel C terminus is reported in Sartorius et al. (2006).

The genetic association to schizophrenia at the GRM3 locus is intragenic, from a region around exon 3 (Egan et al., 2004; Harrison et al., 2008; Schizophrenia Working Group of the Psychiatric Genomics Consortium, 2014). In the absence of evidence for coding variants in linkage disequilibrium, the mechanism of genetic association likely involves regulation and expression of the gene, perhaps via an effect on alternative splicing (Xiao et al., 2017). Supporting this contention, Sartorius et al. (2006) identified an mRNA isoform which lacked exon 4 (GRM3Δ4) and which was predicted to encode a truncated protein with a novel 96-amino acid C terminus (mGlu3Δ4; Figure 1). These authors later reported that the relative abundance of GRM3Δ4 mRNA was increased in brain tissue from subjects with a GRM3 schizophrenia risk genotype (Sartorius et al., 2008). The isoform may thereby contribute to the roles which GRM3/mGlu3 plays in the disorder. Sartorius et al. (2006) showed that GRM3Δ4 is translated and localizes to membranes and, in neurons, to neurites. However, nothing is known about whether mGlu3Δ4 is functional, either in its own right or by interacting with full-length mGlu3. Precedents for both possibilities, especially the latter, are provided by other GPCRs (Niswender and Conn, 2010; Wise, 2012). For example, a splice variant can impact on the activity of its canonical receptor via several mechanisms, including altered intracellular trafficking, heterodimerization and effects on ligand binding. The goal of this study was to investigate these processes, using a range of methods and assays, in HEK293T/17 cells transiently transfected with GRM3 and/or GRM3Δ4.

## Materials and methods

### Constructs

The open reading frames for GRM3, GRM3Δ4, and GRM3Δ4 with a C-terminal V5 tag (GRM3Δ4-V5) were cloned into the pCI-neo expression vector (Promega E1841) using *EcoR1* and *XbaI* restriction sites. This vector uses the human cytomegalovirus promoter to drive constitutive expression in mammalian cells. Constructs were sequenced and corrected by site-directed mutagenesis (Stratagene 200523) prior to use for transfection of human embryonic kidney (HEK293T/17) cells. This cell line was chosen since it does not express endogenous mGlu3, confirmed by reverse transcription polymerase chain reaction (data not shown).

### Cell culture and transient transfection

HEK293T/17 cells (ATCC CRL-11268) were maintained in Dulbbeco’s modified Eagle’s medium (DMEM; Sigma D6546), supplemented with 10% foetal bovine serum (FBS) (Sigma F9665) and 4 mM l-glutamine (Sigma G7513). Cells were grown on 3.8 cm^2^ glass coverslips for immunocytochemistry, and in flasks for western blot and radioligand binding assays, at a seeding density of 5 × 10^4^ cells/cm^2^.

For transfection, cells were seeded, cultured for 24 h and then transfected using a standard lipid protocol. Briefly, each construct (at a concentration of 533.33 ng/μL equating to 200 ng/cm^2^) was mixed with 20% glucose in a ratio of 3:1 DNA to glucose. Polyethylenimine (PEI; Sigma-Aldrich 408727) at a concentration of 5.6 mg/mL was added to the mix at a ratio of 1:3.3 (PEI to DNA glucose). The mixture was incubated for 5 min at room temperature and then added to transfection culture media (DMEM 4.5 g/L glucose, 10% FBS and 2 mM glutamine). Cells were incubated in transfection mix for 24 h, following which, the media was exchanged and cells were incubated for a further 24 h before harvesting.

### Membrane and cytosolic fraction preparation

Extraction of a cellular fraction enriched for membranes was performed using a kit (Biovision Incorporated, Milipitas, California, USA), according to the manufacturer’s instructions, with minor modifications. Cells were harvested with a cell scraper, and lysed in homogenization buffer using a dounce homogenizer. For western blot experiments, 100 µM iodoacetamide and protease inhibitors (cOmplete^TM^, Roche) were added to this buffer. Lysed cells were centrifuged at 1000 × *g* for 10 min at 4°C. The resultant supernatant was collected and centrifuged at 10,000 × *g* for 30 min at 4°C to pellet the membrane fraction, with the final supernatant becoming the cytosolic fraction. For western blot assays, the pellet was re-suspended in RIPA buffer (with added protease inhibitors) and for radioligand binding experiments it was re-suspended in phosphate buffer (10 mM K_2_HPO_4_, 1 mM KH_2_PO_4_ and 100 mM KBr; pH 7.6). Total protein concentration was determined using the Bradford assay (Sigma B6916) following standard protocols.

### Western blotting

Western blot experiments were carried out as previously described (García-Bea et al., 2016). Briefly, 1 µg total membrane protein was run on 4–20% mini-Protean polyacrylamide gel (Bio-Rad 4561095), in SDS/Tris/glycine buffer (25 mM Tris-HCl, 250 mM glycine, 0.1% SDS) at 100V for 2 h. Proteins were transferred to a PVDF (polyvinylidene difluoride) membrane (25 V overnight) and blocked with 5% skimmed milk in PBST (phosphate buffer containing 0.1% tween 20) for 40 min. The primary and secondary antibody incubations were performed at room temperature in PBST with 2% skimmed milk, for 1 h and 40 min respectively. Enhanced chemiluminescence reagent (GE Healthcare, Fisher Scientific, Loughborough, UK) was added as per the manufacturer’s instructions. The blots were then exposed to film (GE Healthcare) and digitally captured using an AlphaImager3400 system. Details of the antibodies used are given in Table 1.

**Table 1.** Details of antibodies and concentrations used.

Antibody	Supplier	Product code	Batch	Epitope	Concentration used		
					WB	ICC	Co-IP
mGlu3	Abcam	ab166608	YJ100911CS	845-C terminus	1:100,000	1:5000	–
mGlu3	Abcam	ab188750	GR230043-1, GR230043-2	N-terminal domain	1:1000		
V5 HRP conjugated	Invitrogen	46-0708	861217	V5	1:10,000	–	–
V5 FITC	Invitrogen	46-0308	1819586	V5		1:1000	
mGlu2/3	Millipore	AB1553	1652168	C-terminus with glutaraldehyde (NGREVVDSTTSSL)	–	–	1:50
N-cadherin	BD Biosciences	610920	78545	–	1:10,000	–	–
GAPDH	Abcam	ab9484	GR165366-3	–	1:5000	–	–
α-tubulin	Abcam	ab7291	GR200985-3	aa 426–450	–	1:2000	–
HRP goat anti-rabbit	Biorad	172-1019	350003011	–	1:10,000	–	–
HRP goat anti-mouse	Biorad	172-1011	350003068	–	1:5000	–	–
Alexa 568 goat anti- rabbit	Invitrogen	A11011	623962	–	–	1:1000	–
Alexa 488 goat anti-mouse	Invitrogen	A11001	632115	–	–	1:1000	–
Alexa 488 donkey anti-mouse	Invitrogen	A10037	1696197	–	–	1:1000	–

Co-IP: co-immunoprecipitation; FITC: fluorescein isothiocyanate; HRP: horseradish peroxidase; ICC: immunocytochemistry; WB: western blot; aa: amino acids.

### Immunocytochemistry

HEK293T/17 cells were grown on glass coverslips coated with 50 µg/mL poly-l-lysine (Sigma) and transfected as described above. Cells were fixed in paraformaldehyde (4% w/v in phosphate buffered saline (PBS)) for 15 min at room temperature, washed three times for 5 min in PBS. Cells were blocked and permeabilized in PBS containing 0.1% Triton-100X and 10% goat serum at room temperature for 40 min. Primary antibodies (Table 1) were diluted in the blocking solution and incubated with cells for 1 h at room temperature. Coverslips were washed three times in PBS for 15 min, incubated with secondary antibodies (Table 1) for 40 min at room temperature and washed three times in PBS for 15 min, with a final rinse in distilled water. Coverslips were air dried, mounted in Vectashield Antifade Mounting Medium with DAPI (Vector, H-1200) and stored at 4°C.

### Co-immunoprecipitation

Co-immunoprecipitation studies were performed as previously described (González-Maeso et al., 2008) with minor modifications. Briefly, 1 mg total membrane protein was incubated for 1 h at 4°C under rotation with equal volume of RIPA buffer (R0278, Sigma) containing 1% SDS and 2% Triton X-100. After centrifugation at 17,000 x g for 15 min at 4°C, supernatant was incubated for an hour with protein A/G beads (sc-2003, Santa Cruz) and then centrifuged at 14,000 rev/min for 1 min. Four hundred microlitres of the supernatant was incubated overnight with 40 µL of protein A/G beads and 1:50 dilution of mGlu2/3 antibody (Table 1) at 4°C on a rotation wheel. The sample was centrifuged for 1 min at 17,000 x g at 4°C, precipitated beads were washed three times with RIPA buffer containing protease inhibitors (cOmplete^TM^, Roche) and re-suspended in RIPA buffer containing 0.5% SDS and 1% Triton X-100. Beads were diluted with 2× Laemmli buffer, boiled for 5 min, centrifuged at 17,000 x g for 1 min at 4°C, the supernatant used in a western blot assay and probed using a V5 tag antibody (Table 1).

### Radioligand binding

[^3^H]LY341495 binding (ART1439 250, American Radiolabeled Chemicals) assays were carried out as previously described with minor modifications (González-Maeso et al., 2008). Briefly, 3 µg total membrane protein was incubated at 4°C with increasing concentrations of [^3^H]LY341495 (0–30 nM) in phosphate buffer (10mM K_2_HPO_4_, 1 mM KH_2_PO_4_ and 100 mM KBr; pH 7.6, also referred to as incubation buffer). After 90 min, free radioligand was separated from bound radioligand by rapid filtration under vacuum through GF/C glass fibre filters (Whatman, 1822-849) pre-wetted with incubation buffer. Filters were then washed three times with cold incubation buffer and counted for radioactivity (5 min) by liquid scintillation spectrometry using Ecoscint H scintillation solution (National Diagnostics) and a Tri-Carb analyser (PerkinElmer) with a counting efficiency of 65% for [^3^H]. Non-specific binding was determined in the presence of 1 mM l-glutamic acid. All samples were run in duplicate for each experiment. Experiments for each condition were repeated a minimum of three times.

### Statistical analysis

Statistical comparisons of mGlu3 immunoreactivity in western blots of cells expressing GRM3 alone or GRM3 plus GRM3Δ4 were conducted by unpaired Student’s *t*-test. Values for apparent equilibrium dissociation constant (Kd) and for maximum density of specific binding sites (Bmax) in saturation binding assays were determined by non-linear analysis. Comparisons of Bmax and Kd between groups were conducted by one-way analysis of variance (ANOVA) followed by a Tukey post-hoc test. All statistical analyses were performed using GraphPad Prism, v. 6.0. The level of significance was set at *p*<0.05, two-tailed.

## Results

### mGlu3Δ4 exists in monomeric and dimeric forms and localizes primarily to cell membranes

**Figure 2.**
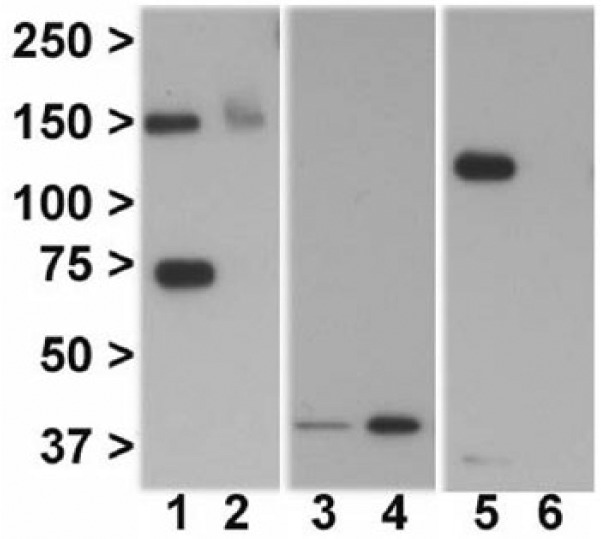
Immunoblotting shows mGlu3Δ4 is enriched in the membrane fraction and is present as a dimer as well as monomer. Lane 1: mGlu3Δ4, membrane fraction. Lane 2: mGlu3Δ4, cytosolic fraction. Lane 3: GAPDH, membrane fraction. Lane 4: GAPDH, cytosolic fraction. Lane 5: N-cadherin, membrane fraction. Lane 6: N-cadherin, cytosolic fraction. Lanes 1, 3 and 5, and lanes 2, 4 and 6 are from the same sample.

In HEK-293T/17 cells transfected with GRM3Δ4, western blotting with an N-terminal anti-mGlu3 antibody revealed bands at ~75kDa and ~150kDa, corresponding to the predicted molecular weights for monomeric and homodimeric mGlu3Δ4 respectively (Figure 2, lanes 1 and 2). Signal was concentrated in the membrane fraction (Figure 2, lane 1), with much weaker immunoreactivity, only clearly detectable for the dimer, in the cytosolic fraction (Figure 2, lane 2). The same banding pattern and predominant membrane localization for mGlu3Δ4 was seen following transfection of GRM3Δ4-V5 (data not shown), and when using a custom anti-mGlu3Δ4 antibody reported previously (García-Bea et al., 2016; data not shown). The cytosolic protein GAPDH (Figure 2, lanes 3 and 4) and the membrane protein N-cadherin (Figure 2, lanes 5 and 6) were used to assess the purity of each fraction.

**Figure 3.**
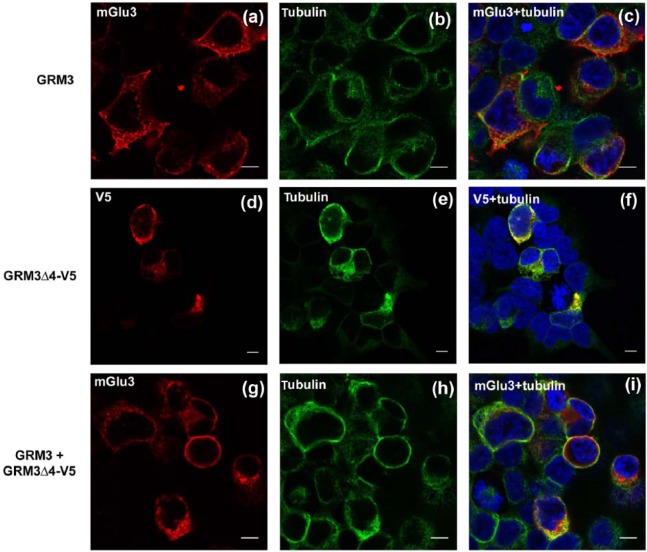
Immunofluorescence reveals predominant membrane localization of mGlu3 and mGlu3Δ4. (a)–(c) Transfection of GRM3, showing immunoreactivity for mGlu3 (a), tubulin (b) and merged image (c). (d)–(f) Transfection of GRM3Δ4-V5, showing immunoreactivity for mGlu3Δ4 (d), tubulin (e) and merged image (f). (g)–(i) Co-transfection of GRM3 and GRM3Δ4-V5, showing immunoreactivity of mGlu3 (g), tubulin (h) and merged image (i). DAPI staining (blue) is shown in (c), (f) and (i). Bar: 10µm (note different magnification in (d)–(f)).

Immunofluorescence was carried out to provide further information about the cellular localization of mGlu3Δ4 (Figure 3). First, we confirmed that after transfection of GRM3, canonical mGlu3 showed the expected localization to plasma membrane and other membranes (Figure 3(a)), corroborated by co-immunostaining with alpha-tubulin to delineate cell morphology (Figure 3(b) and (c)). Separate transfection of GRM3Δ4-V5 showed a similar distribution for mGlu3Δ4 (Figure 3(d) to (f)), with signal concentrated over membranes including the plasma membrane. Co-transfection of GRM3 and GRM3Δ4-V5 did not appear to alter the distribution of mGlu3 immunoreactivity (Figure 3(g) to (i). The same intracellular distribution of mGlu3Δ4 was seen when non-tagged GRM3Δ4 was singly transfected and detected using the N-terminal anti-mGlu3 antibody, indicating that the V5 tag is not affecting trafficking (data not shown).

### Co-immunoprecipitation shows mGlu3Δ4 interaction with mGlu3

**Figure 4.**
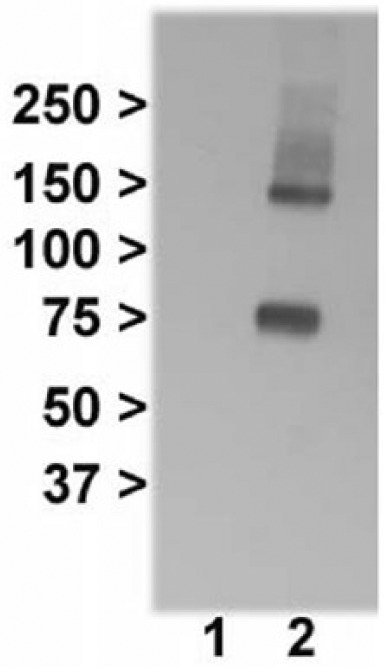
Co-immunoprecipitation shows interaction of mGlu3 and mGlu3Δ4. Lane 1: cells transfected with either GRM3 or GRM3Δ4-V5 were mixed together, followed by immunoprecipitation with the mGlu2/3 antibody and probed with the V5 tag antibody. Lane 2: cells co-transfected with GRM3 and GRM3Δ4-V5, followed by immunoprecipitation with the mGlu2/3 antibody and probed with the V5 tag antibody.

To see whether mGlu3Δ4 and mGlu3 interact, we used co-immunoprecipitation, with an mGlu2/3 antibody (Table 1) for pull down, and immunoblotting with the V5 tag antibody. As expected, no bands were seen when cells were separately transfected with GRM3Δ4-V5 and GRM3 then mixed together (Figure 4, lane 1), whereas cells co-transfected with GRM3Δ4-V5 and GRM3 produced bands of the predicted size for mGlu3Δ4 (Figure 4, lane 2), showing that mGlu3 does physically interact with mGlu3Δ4.

### mGlu3Δ4 reduces [^3^H]LY341495 binding to mGlu3 and the membrane abundance of mGlu3

**Figure 5.**
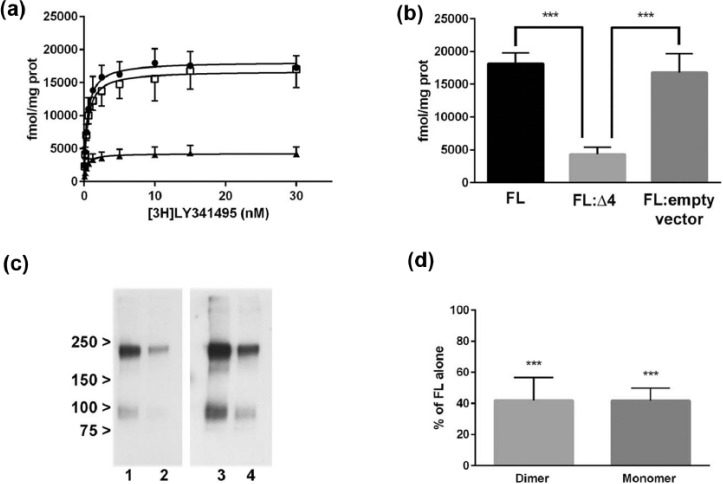
mGlu3Δ4 impacts on [^3^H]LY341495 binding to, and abundance of, mGlu3. (a) Saturation curves show binding of [^3^H]LY341495 after transfection of full-length GRM3 alone (circles), transfection of GRM3 and empty vector (squares) and co-transfection of GRM3 and GRM3Δ4 (triangles). (b) [^3^H]LY341495 Bmax for full-length GRM3 (FL), co-transfected GRM3 and GRM3Δ4 (FL:Δ4), and full length GRM3 with empty vector (FL: empty vector). ***p<0.001. (c) Western blots of membrane protein probed with mGlu3 C-terminal antibody (Table 1). Lane 1: full-length GRM3 transfected alone. Lane 2: co-transfection of GRM3 and GRM3Δ4. Note lower immunoreactivity in 2 compared with 1. Lanes 3 and 4 are longer exposures of lanes 1 and 2 to improve visualization of the ~95kDa monomer band. (d): relative quantitation of western blots after co-transfection of GRM3 and GRM3Δ4 showing reduced immunoreactivity for mGlu3 dimer and monomer compared with immunoreactivity after transfection of GRM3 alone. ***p<0.001. prot: protein

As an initial measure of receptor function, we examined binding of the selective mGlu2/3 antagonist [^3^H]LY341495 (Johnson et al., 1999; Kingston et al., 1998). We found no specific binding in cells which had been transfected with GRM3Δ4 (data not shown), whereas the radioligand bound to GRM3-transfected cells with an estimated Bmax of 18,150±317 fmol/mg protein and Kd 0.43±0.04 nM (Figure 5(a)). Co-transfection of GRM3Δ4 with GRM3 substantially reduced Bmax (Figure 5(a) and (b)) but did not alter Kd (0.34±0.06 *vs*. 0.43±0.04 nM). Bmax was unaffected by co-transfection of GRM3 with empty vector (Figure 5(a) and (b)).

We then used protein extracts from the radioligand binding experiments for western blots, and found that mGlu3 immunoreactivity was reduced in the membranes of cells co-transfected with GRM3 and GRM3Δ4 compared with cells transfected with GRM3 alone (Figure 5(c) and (d)), suggesting that the decreased binding to mGlu3 in the presence of mGlu3Δ4 reflected decreased abundance of mGlu3. The reduced membrane mGlu3 immunoreactivity after co-transfection was not accompanied by any compensatory increase in cytoplasmic mGlu3 immunoreactivity (data not shown).

## Discussion

A transcript encoding a C-terminal variant isoform of mGlu3, GRM3Δ4, was previously identified (Sartorius et al., 2006) and its expression found to be influenced by a schizophrenia-associated GRM3 risk SNP (Sartorius et al., 2008). Here we show, using HEK293T/17 cells, that this transcript is translated, with the resulting mGlu3Δ4 protein concentrated in membranes, including the plasma membrane (Figures 2 and 3), confirming earlier observations (García-Bea et al., 2016; Sartorius et al., 2006). We further show that mGlu3Δ4 co-immunoprecipitates with mGlu3 (Figure 4) and its presence decreases the abundance of canonical mGlu3 and the Bmax of the mGlu2/3 antagonist [^3^H]LY341495 (Figure 5). Thus, mGlu3Δ4 appears to serve as a negative modulator of mGlu3, and in this way the GRM3Δ4 isoform may contribute to the mechanism by which allelic variation in GRM3 is associated with schizophrenia risk. The findings are also relevant to investigations of mGlu3 ligands as potential therapeutic agents in a range of diseases.

GPCRs are thought to function largely if not entirely as dimers rather than as monomers (Gurevich and Gurevich, 2008). As expected, we detected full-length mGlu3 primarily as a dimer (as judged by molecular weight) (Figure 5(c); see also García-Bea et al., 2016). Interestingly, mGlu3Δ4 was also detected partly as a dimer (Figure 2, lane 1), even though it lacks the transmembrane domains thought to be critical to GPCR dimerization (Bulenger et al., 2005). Presumably either the conserved N-terminal region or the novel C terminus is sufficient. Our co-immunoprecipitation data show convincingly that mGlu3 and mGlu3Δ4 interact physically and imply, but do not prove, the occurrence of heterodimerization (Scarselli et al., 2016). This needs further study, given the potential physiological and therapeutic significance of heterodimers (Farran, 2017; Milligan, 2009; Rozenfeld and Devi, 2010).

Originally thought to be rare, it is now clear that most GPCRs, including mGlu receptors, are expressed as splice variants in addition to the canonical (wild-type) receptor (Einstein et al., 2008; Niswender and Conn, 2010). Many of these isoforms, like mGlu3Δ4, are truncated (albeit to a less extreme degree), with loss of one or more transmembrane domains and with a novel C terminus (Wise, 2012). The significance of many of these GPCR variants is unknown; even their existence at the protein rather than mRNA level has not always been established. However, there are some notable examples. The dopamine D3 receptor exists as a truncated variant, D3nf, with which the full-length D3 receptor interacts, and the presence of D3nf prevents D3 receptor localization to the plasma membrane (Karpa et al., 2000). Indeed, many truncated variants are thought to promote retention of the canonical receptor in the endoplasmic reticulum, thereby acting in a dominant negative fashion (Bulenger et al., 2005; Wise, 2012). Our data indicate that mGlu3Δ4 may also act partially in this way since, although there was no qualitative change in the membrane localization of mGlu3 in the presence of mGlu3Δ4 as observed using immunofluorescence (Figure 3), there was a reduction in membrane-bound mGlu3 immunoreactivity as assessed using semi-quantitative western blots (Figure 5(c) and (d)). It is tempting to link the latter observation to the finding that co-transfection with GRM3Δ4 led to reduced [^3^H]LY341495 binding to mGlu3 (Figure 5(b)). That is, Bmax is lower because fewer mGlu3 receptors are available for binding; however, this remains to be demonstrated directly. Similarly, whether these effects result directly from the physical interaction between the proteins indicated by the co-immunoprecipitation data awaits investigation. One possibility is that mGlu3Δ4 alters the processing of mGlu3, perhaps promoting lysosomal degradation rather than membrane insertion or recycling; such effects have been reported for C-terminal variation in other GPCRs (Heydorn et al., 2004; Trejo and Coughlin, 1999). However, a search of the UniProt database using ‘blastp’ does not reveal any homologies or motifs within the amino acid sequence of the mGlu3Δ4 C terminus, which might shed light on this or other putative mechanisms; the search merely shows that a similar mGlu3 variant is found in several other mammalian species.

Another question pertaining to GPCR splice variants is whether they are functional in their own right in the sense of binding cognate ligand and initiating downstream signalling. Many do not appear to be active, but several such examples have been identified, including the mu opioid receptor MOR-1, somatostatin sst5 receptor and chemokine receptors CCR5 and CXR4 (Wise, 2012). Several active C-terminal splice variants of mGlu1 are also known, which retain varying degrees of agonist potency and second messenger responses (Hermans and Challiss, 2001). However, we did not find any evidence that mGlu3Δ4 could bind [^3^H]LY341495. This may be because the N-terminal binding domain is truncated (Figure 1(b)), and mGlu3Δ4 is also missing critical cysteine residues adjacent to the transmembrane domain which stabilize the conformation required for ligand binding (Huang et al., 2011; Muto et al., 2007; Vafabakhsh et al., 2016). It may also be relevant that the C-terminus of mGlu3Δ4 lacks a 20 amino acid region which serves as a protein phosphatase PP2C binding domain within mGlu3, given that dephosphorylation is thought to influence signalling and trafficking of the receptor (Flajolet et al., 2003).

In summary, we found that mGlu3Δ4, an isoform of mGlu3 which is associated with genetic risk for schizophrenia, impacts upon the functioning of canonical mGlu3: mGlu3Δ4 decreases the availability of mGlu3 to bind ligand, potentially reducing signalling through the receptor. This effect has therapeutic relevance in that it may modify the effect of mGlu3 agonists, antagonists and allosteric modulators. However, further work is required to extend the findings and clarify these interpretations. First, to corroborate our data using more physiological levels of mGlu3 and mGlu3Δ4 expression, and using neuronal or glial cell lines, or cells of these lineages derived from stem cells – although in these cells experiments will be complicated by endogenous expression of the receptor. Second, to elucidate the molecular basis for the interactions between mGlu3 and mGlu3Δ4, and the downstream consequences for mGlu3-mediated signalling and for interactions of mGlu3 with other proteins (Magalhaes et al., 2012). Third, the presence of endogenous mGlu3Δ4 in human brain remains uncertain. Sartorius et al. (2006) reported a band of the predicted size of the isoform in homogenates of frontal cortex, but García-Bea et al. (2016) were unable to detect it. The discrepancy between the studies may relate to the fact that they used different antibodies and investigated different brain regions. Finally, gene expression including alternative splicing is increasingly recognized to be an important contributor to genetic associations with schizophrenia (Fromer et al., 2016; Harrison and Weinberger, 2005; Kleinman et al., 2011; Law et al., 2006; Li et al., 2016a; Takata et al., 2017; Tao et al., 2014) and other diseases (Li et al., 2016b; Parikshak et al., 2016). Most empirical studies investigating this topic focus on mRNA because of the availability of sensitive and specific methods for transcript identification and quantification. However, it is also necessary to determine the downstream effects on encoded protein isoforms, as illustrated here, in order to understand fully the pathophysiological and therapeutic implications of the genomic findings (Collier et al., 2016; Harrison, 2015; Schubert et al., 2015).
